# Culture Medium Enriched with Ultrafine Carbon Monoxide Bubbles Enhances In Vitro Blastocyst Formation of In Vivo-Fertilized Mouse Zygotes

**DOI:** 10.3390/antiox14060684

**Published:** 2025-06-04

**Authors:** Toyofumi Hirakawa, Kazuhiko Nakabayashi, Noriko Ito, Keisuke Ishiwata, Daichi Urushiyama, Kohei Miyata, Tsukasa Baba, Kenichiro Hata, Shin’ichiro Yasunaga, Fusanori Yotsumoto, Katsuro Tachibana, Shingo Miyamoto

**Affiliations:** 1 Department of Obstetrics & Gynecology, Faculty of Medicine, Fukuoka University, Fukuoka 8140180, Japan; thira@fukuoka-u.ac.jp (T.H.); duru@fukuoka-u.ac.jp (D.U.); kmiyata@outlook.com (K.M.); yotsumoto@cis.fukuoka-u.ac.jp (F.Y.); 2 Department of Maternal-Fetal Biology, National Center for Child Health and Development, Tokyo 1578535, Japan; nakabaya-k@ncchd.go.jp (K.N.); ito-nor@ncchd.go.jp (N.I.); ishiwata-k@ncchd.go.jp (K.I.); hata-k@ncchd.go.jp (K.H.); 3 Department of Obstetrics & Gynecology, School of Medicine, Iwate Medical University, Morioka 0283695, Japan; babatsukasa@gmail.com; 4 Department of Biochemistry, Faculty of Medicine, Fukuoka University, Fukuoka 8140180, Japan; syasunag@fukuoka-u.ac.jp; 5 Department of Anatomy, Faculty of Medicine, Fukuoka University, Fukuoka 8140180, Japan; k-tachi@fukuoka-u.ac.jp

**Keywords:** gasotransmitter, carbon monoxide, ultrafine bubble, blastocyst

## Abstract

Oxidative stress induced by in vitro culture conditions impedes the differentiation of fertilized zygotes. Gasotransmitters containing carbon monoxide (CO) exhibit antioxidant properties when exogenously administered at appropriate concentrations. In this study, CO was incorporated into ultrafine bubbles (UFBs) to devise an innovative method for promoting the efficient differentiation of fertilized mouse zygotes into blastocysts within an in vitro culture environment. While CO typically dissipates rapidly in culture media, its encapsulation into UFBs enabled its prolonged retention within the medium. Fertilized mouse zygotes cultured in the UFB medium exhibited a significantly higher rate of blastocyst hatching compared to those cultured in conventional media. Furthermore, Gene Ontology analysis revealed elevated expression of mitochondrial-related genes and genes essential for blastocyst maturation in the UFB culture medium. These findings underscore the potential of CO-UFB as a potent agent for improving in vitro blastocyst formation and hatching by mitigating oxidative stress, thereby offering a promising strategy for enhancing assisted reproductive technologies.

## 1. Introduction

Birthrates are steadily declining in many developed countries, with infertility notably increasing [1]. Primary therapeutic approaches for infertility encompass assisted reproductive technologies, including timed intercourse, ovulation induction, artificial insemination, and in vitro fertilization (IVF). When pregnancy is not achieved through initial treatment modalities, a transition to more advanced interventions is recommended as necessary. The most sophisticated treatments include IVF and intracytoplasmic sperm injection (ICSI), wherein oocytes are retrieved from the ovaries, fertilized with sperm in vitro, and subsequently cultured to form blastocysts suitable for implantation before being transferred back into the uterus. Excessive production of reactive oxygen species (ROS) by preimplantation embryos during in vitro culture exerts deleterious effects, diminishing the differentiation potential of fertilized zygotes [2]. In vivo, a delicate balance between ROS and antioxidants is physiologically maintained, whereas in vitro culture disrupts this equilibrium, leading to increased oxidative stress [3]. The endogenous antioxidant system in vivo safeguards embryos from oxidative damage; however, oxidative stress caused by ROS accumulation in embryos generated in vitro compromises embryogenesis by inducing apoptosis, thereby reducing fertilization rates and hindering blastocyst formation [4,5]. The present study, as well as most of the previous research cited herein, was conducted using mouse embryos or murine-derived materials. Recent studies have highlighted the critical role of ROS generation within and around embryonic cells during in vitro culture. The regulation and scavenging of ROS are essential for germ cell survival, embryonic development, and successful cryopreservation [6]. Consequently, the incorporation of antioxidants into IVF culture media to mitigate oxidative stress has been reported to enhance preimplantation embryo development [7,8,9,10]. Nevertheless, the efficacy of such interventions remains inconclusive, underscoring the urgent need for novel strategies to optimize in vitro culture conditions and enhance the differentiation potential of fertilized zygotes.

Gasotransmitters are endogenously synthesized gaseous molecules that act as signaling mediators for neurotransmitters, target enzymes, ion channels, and various transporters. Nitric oxide (NO) was the first gasotransmitter identified, recognized for its critical physiological roles [11]. Subsequently, carbon monoxide (CO) and hydrogen sulfide (H_2_S) also garnered attention for their physiological significance, despite their initial classification as toxic substances [12,13]. More recently, all three endogenous gasotransmitters (NO, CO, and H_2_S) have been shown to activate numerous cytoprotective mechanisms. These mechanisms include vasodilation, inhibition of apoptosis, regulation of mitochondrial respiration, activation of antioxidant pathways, and suppression of inflammation, mediated through the modulation of various signaling pathways by specific enzymes [14]. Among these, CO has demonstrated significant potential in mitigating the pathophysiological effects of oxidative stress-related diseases [15,16]. However, the effective administration of CO in cell culture remains challenging owing to its exceptionally short half-life in culture media [17]. To address this, carbon monoxide-releasing molecules (CORMs) have been developed to release CO under specific biological conditions. While CORMs hold promise, their composition—often incorporating metal complexes such as ruthenium, iron, or manganese—poses potential risks of metal-induced cytotoxicity when administered to cells [18,19].

Ultrafine bubbles (UFBs) provide a mechanism for stabilizing and maintaining gaseous substances within liquids without the incorporation of metal complexes. UFBs are defined as bubbles with a volume-equivalent diameter of less than 1 µm [20]. Unlike conventional larger bubbles, which rapidly rise to the surface and dissipate, UFBs exhibit remarkable stability, persisting in liquids for months [21,22]. Leveraging this unique property, various gases have been encapsulated in UFBs, and their applications have been extensively explored across diverse fields, including cleaning [23,24], agriculture [25,26], fisheries [27], medicine [28,29], and pharmaceuticals [30]. Numerous methods for generating UFBs have been reported, such as pressurized dissolution [31], swirling liquid flow [32], and ultrasonic irradiation [22,33]. Among these techniques, the selection of an approach that allows for the preparation of UFB-containing cell culture media while preventing temperature elevation is crucial to avoid denaturation of the medium.

In this study, we utilized fertilized zygotes culture medium infused with UFBs containing CO—a gasotransmitter—to efficiently deliver CO to fertilized zygotes in culture. The findings suggest that this approach enhances differentiation rates by facilitating intracellular activity. This innovative culture method holds potential for improving in vitro culture conditions and blastocyst differentiation rates, ultimately contributing to more efficient embryo transfer and successful outcomes.

## 2. Materials and Methods

### 2.1. Preparation of UFB Culture Medium

Single-Step culture medium (SSCM; Kitazato Corporation Ltd., Shizuoka, Japan) was utilized as the culture medium for mouse embryos. SSCM was chosen due to its established utility in mouse embryo culture and its stable, defined composition. Additionally, because SSCM is also used in human ART, its use in this study was intended to support potential translational applications. The UFB generation system employed was the HMB-H0150 + P001 (TOSSLEC Corporation Ltd., Kyoto, Japan), which operates via ultrasonic irradiation, as previously described [22]. A distinguishing feature of this device is its integrated cooling mechanism, which prevents temperature elevation during UFB generation, thereby mitigating the risk of culture medium denaturation caused by heat. During the UFB generation process, the temperature of the culture medium was maintained below 20 °C through chiller-controlled circulation within the cooling tank. The carbon monoxide-ultrafine bubble (CO-UFB) medium was prepared using CO gas supplied by Fukuoka Oxygen Corporation Ltd. (Fukuoka, Japan). The prepared CO-UFB medium was stored under sterile conditions in 20 mL bottles at 4 °C to ensure stability and integrity.

### 2.2. Nanoparticle Tracking Analysis (NTA) Measurement of UFB

The quantity of nanoparticles in the CO-UFB medium was analyzed using nanoparticle tracking analysis (NTA) with a NanoSight LM10 system (NanoSight, Malvern, UK). The optimal particle count was set to approximately 20–100 particles per frame to ensure accurate tracking and statistically meaningful results, as recommended for NanoSight LM10 analysis. This approach is consistent with that of our previous study [34]. The instrument settings were configured based on the guidelines provided in the NanoSight LM10 User Manual. The camera level was set to level 8, ensuring all particles were clearly visible without exceeding a particle signal saturation threshold of 20%. Autofocus adjustments were made to eliminate blurry particles. Following video capture, the data were processed using NanoSight Software NTA 3.0. Each sample underwent independent analysis a minimum of five times, and the mean values and standard deviations were subsequently calculated.

### 2.3. In Vitro Culture of Embryos

Frozen 2-cell embryos incubated with SSCM without the UFB were used as controls. The CO-UFB medium was diluted with SSCM to prepare various concentration gradients. While the undiluted medium was designated as CO-UFB (1×), the 4-fold, 10-fold, 20-fold, 50-fold, and 100-fold dilutions were labeled as CO-UFB (4×), CO-UFB (10×), CO-UFB (20×), CO-UFB (50×), and CO-UFB (100×), respectively. These multiple concentrations were initially tested to determine the optimal condition for promoting embryo development. SSCM filtered through a 0.22-μm membrane (Millex GV, Millipore Corp., Burlington, MA, USA) was defined as F-Control, and CO-UFB (20×), which was selected for subsequent analysis based on its promising performance and was subjected to the same filtration process and designated as F-CO-UFB.

Embryos were cultured in 50-μL droplets of medium under mineral oil, with 10 embryos placed per droplet. Each experimental group included a total of 150 embryos, divided across three biological replicates. Frozen 2-cell embryos were cultured in 50 µL of either control medium or CO-UFB medium at 37 °C under 5% CO_2_ conditions on Day 0. The embryos, sourced from the Biosafety Research Center, Inc. (Hyogo, Japan), were monitored for blastocyst formation and hatching on Day 4. Morphological assessments of the mouse embryos were conducted sequentially, from the 2-cell stage through to the hatching stage.

### 2.4. cDNA Library Preparation and RNA Sequencing of Blastocysts

Blastocyst samples were obtained on Day 4 from both the control and CO-UFB (20×) groups (*N* = 8 per group), as previously documented [34]. In summary, sequencing libraries for each blastocyst were prepared using the NEBNext Single Cell/Low Input RNA Library Prep Kit for Illumina and the NEBNext Multiplex Oligos for Illumina (96 Unique Dual Index Primer Pairs) (New England BioLabs, Ipswich, MA, USA), following the manufacturer’s guidelines to generate cDNA from cells as the starting material. The libraries were subsequently sequenced on the Illumina HiSeq X platform (Illumina, San Diego, CA, USA) with paired-end 151 bp reads and dual indexing.

### 2.5. RNA-Sequencing Data Analysis of Blastocysts

Genetic analysis of the blastocysts was conducted following previously established methodologies [34]. In brief, raw sequencing reads from 16 RNA sequencing libraries, each containing between 15.0 and 22.4 million read pairs, were processed for transcript quantification using Salmon 1.5.2 (https://salmon.readthedocs.io/en/latest/salmon.html, accessed on 29 April 2025) [35]. For Salmon’s mapping-based mode, a transcriptome index was generated from gencode.vM23.transcripts.fa.gz, with the complete human genome (GRCm38.primary_assembly.genome.fa.gz) serving as the decoy sequence. The quant.sf data from all 16 samples were consolidated into a single dataset using the R package tximport version 1.20.0. [36]. Subsequent analysis of count data for 54,281 gene IDs was performed with the R package EdgeR version 4.4.2. [37], which included filtering, normalization, clustering, and identification of differentially expressed genes (DEGs) between conditions, applying a significance threshold of FDR q-value < 0.05. Gene ontology analysis of the identified DEGs was conducted using Metascape [38].

### 2.6. Statistical Analysis

Data analysis was conducted using Prism 8 software (GraphPad Software Inc., San Diego, CA, USA). The results are presented as mean ± standard error. Nanoparticle concentrations, blastocyst formation rates, and hatching rates across the different groups were evaluated using the Mann–Whitney U test and the Benjamini–Hochberg method. Statistical significance was determined for *p* values below 0.05.

## 3. Results

### 3.1. Comparison of CO-UFB Size and Abundance

**Figure 1:**
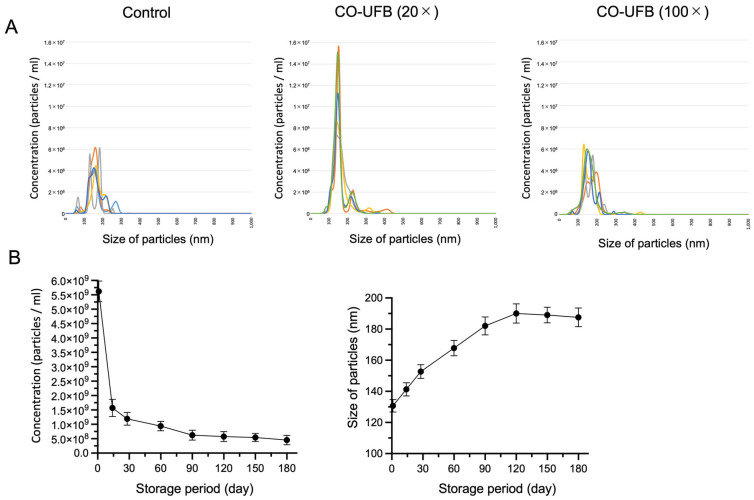
Ultrafine bubble (UFB) measurements. (**A**) Number and distribution of nanoparticles in the culture medium by NanoSight. The different colors represent five independent measurements. (**B**) Fluctuations in the UFB concentration or size in the retention period. Fluctuations in the UFB concentration or size are shown for a period of 6 months. Each circle or bar indicates the mean or standard deviation on days 1, 14, 30, 60, 90, 120, 150, and 180. CO-UFB: carbon monoxide-ultrafine bubble.

In this study, the nanoparticle sizes ranged from 50 to 500 nm, with the majority falling within the 100–200 nm range across all media. The mean size and concentration of nanoparticles in the control group containing nanosites were 160.7 ± 38.3 nm (mean ± SD) and 2.93 × 10^8^ ± 1.37 × 10^7^/mL (mean ± SD), respectively (Figure 1A). The nanosites in the control group likely originated from components of the SSCM culture medium itself, as no UFBs were intentionally introduced. To estimate the concentration of CO-UFB, we utilized CO-UFB (20×) and CO-UFB (100×). In the CO-UFB (20×) group, the mean nanoparticle size and concentration were 160.6 ± 44.8 nm (mean ± SD) and 5.74 × 10^8^ ± 2.47 × 10^7^/mL (mean ± SD), respectively. Meanwhile, in the CO-UFB (100×) group, these values were 164.5 ± 41.4 nm (mean ± SD) and 3.46 × 10^8^ ± 2.33 × 10^7^/mL (mean ± SD), respectively (Figure 1A). Given that the difference in nanoparticle counts between CO-UFB and the control represents the number of UFBs generated, the estimated UFB concentration was approximately 2.81 × 10^8^/mL in the CO-UFB (20×) group and 5.30 × 10^7^/mL in the CO-UFB (100×) group.

To evaluate the decay rate and size evolution of CO-UFB, we monitored nanoparticle concentration and size changes over time in the CO-UFB (1×) group. On the first day post-synthesis, the CO-UFB (1×) concentration was 5.62 × 10^9^ ± 3.60 × 10^8^/mL (mean ± SD), which significantly declined to 1.57 × 10^9^ ± 2.96 × 10^8^/mL (mean ± SD) by Day 14 (Figure 1B). Thereafter, the reduction was not statistically significant, but a gradual decline was observed until day 180 (Figure 1B). Conversely, the size of CO-UFB (1×) exhibited a progressive increase from 130.7 ± 4.0 nm (mean ± SD) on Day 1 to 182.0 ± 5.8 nm (mean ± SD) on Day 90 (Figure 1B). Beyond this point, the size of UFBs showed no substantial expansion over the 180-day period (Figure 1B).

### 3.2. Effect of CO-UFB on Embryonic Development

**Figure 2:**
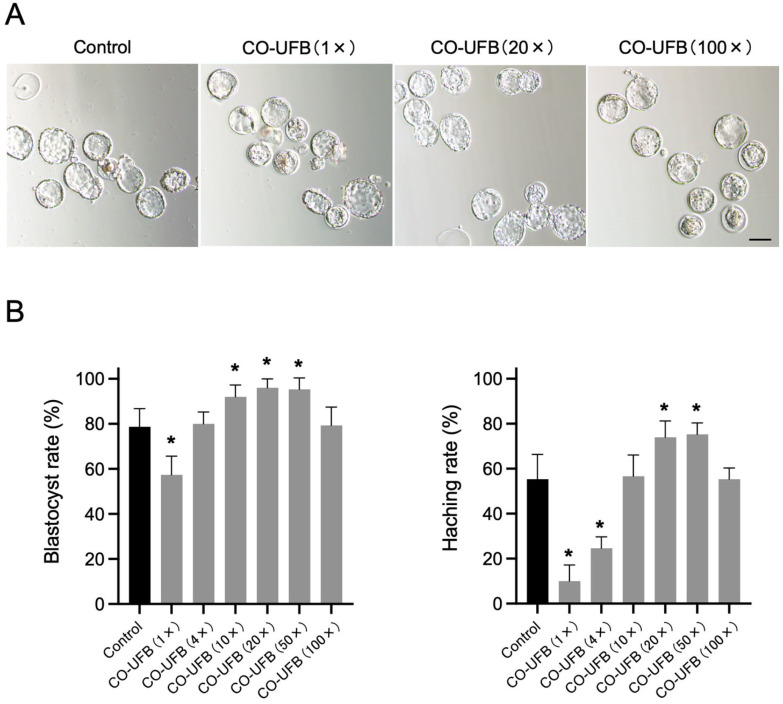
(**A**) Embryo morphology on Day 4. Scale bar = 50 μm. (**B**) Blastocyst and hatching rates of in vitro-produced mouse embryos with CO-UFB medium. The mean percentage was calculated from three independent experiments. *n* = 150 per group. Data are presented as the mean ± standard error. * *p* < 0.05 vs. control. CO-UFB: carbon monoxide ultrafine bubble medium.

In the control group, 78.7 ± 6.6% of 2-cell embryos progressed to the blastocyst stage by Day 4. In comparison, the blastocyst formation rates were 92.0 ± 4.3% in the CO-UFB (10×), 96.0 ± 3.3% in the CO-UFB (20×), and 95.3 ± 4.1% in the CO-UFB (50×) groups (Figure 2). The embryo development rates in the CO-UFB (10×), CO-UFB (20×), and CO-UFB (50×) groups were significantly higher than those observed in the control group (*p* < 0.05) (Figure 2). Conversely, in the CO-UFB (1×) group, only 57.3 ± 6.8% of embryos reached the blastocyst stage, a significantly lower rate than that in the control group (*p* < 0.05) (Figure 2). Regarding blastocyst hatching, 55.3 ± 9.0% of 2-cell embryos in the control group developed into hatched blastocysts by Day 4. In contrast, the hatching rates were 74.0 ± 5.9% in the CO-UFB (20×) group and 75.3 ± 4.1% in the CO-UFB (50×) group, both of which were significantly higher than those in the control group (*p* < 0.05) (Figure 2). However, in the CO-UFB (1×) and CO-UFB (4×) groups, the hatching rates were markedly lower, with only 10.0 ± 5.9% and 24.7 ± 4.1% of embryos developing into hatched blastocysts, respectively (Figure 2). These values were significantly lower than those observed in the control group (*p* < 0.05) (Figure 2). These results indicate that the CO-UFB medium enhances embryonic development and hatching within an appropriate concentration range. Based on the observed blastocyst formation and hatching rates, we estimated the optimal UFB concentration for promoting embryonic development to be approximately 1 × 10^8^ to 6 × 10^8^/mL. In contrast, excessive CO-UFB concentrations appeared to impair embryonic development.

### 3.3. Effect of CO-UFB (Filtration) on Embryonic Development

**Figure 3:**
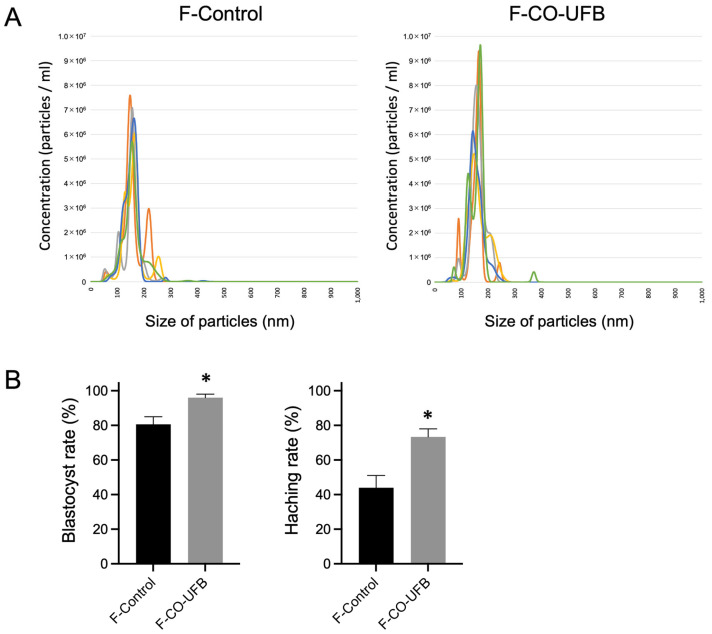
(**A**) Number and distribution of nanoparticles in the culture medium by NanoSight in the culture medium after filtration. The different colors represent five independent measurements. (**B**) Blastocyst and hatching rates of in vitro-produced mouse embryos with F-CO-UFB medium. The mean percentage was calculated from three independent experiments. *n* = 150 per group. Data are presented as the mean ± standard error. * *p* < 0.05 vs. F-Control. F-Control: control medium with filtration. F-CO-UFB: carbon monoxide ultrafine bubble medium with filtration.

Among the tested concentrations, CO-UFB (20×) showed the highest rates of blastocyst formation and hatching. Therefore, this concentration was used in subsequent experiments to evaluate the influence of the bubble size. The mean size and concentration of nanoparticles in the F-Control group, comprising SSCM filtered at 220 nm, were 155.2 ± 22.2 nm (mean ± SD) and 3.33 × 10^8^ ± 1.57 × 10^7^/mL (mean ± SD), respectively (Figure 3A). Similarly, the nanoparticle size and concentration in the F-CO-UFB group, representing CO-UFB (20×) filtered at 220 nm, were 158.9 ± 28.0 nm (mean ± SD) and 3.75 × 10^8^ ± 1.88 × 10^7^/mL (mean ± SD), respectively (Figure 3A). Therefore, the number of UFBs below 220 nm in the F-CO-UFB group is estimated to be approximately 4.3 × 10^7^/mL. In the F-Control group, 80.7 ± 6.2% of 2-cell embryos progressed to the blastocyst stage, with 44.0 ± 9.9% successfully hatching by Day 4 (Figure 3B). In contrast, the F-CO-UFB group exhibited significantly enhanced developmental outcomes, with 96.0 ± 2.8% of embryos reaching the blastocyst stage and 73.3 ± 6.6% undergoing hatching (Figure 3B). The embryo development and hatching rates in the F-CO-UFB group were markedly higher than those observed in the F-Control group (*p* < 0.05) (Figure 3B). These findings suggest that the optimal ultrafine bubble size for encapsulating CO and other bioactive substances in fertilized zygotes cultures is 220 nm or smaller.

### 3.4. Effect of UFB on the Gene of Blastocysts

**Figure 4:**
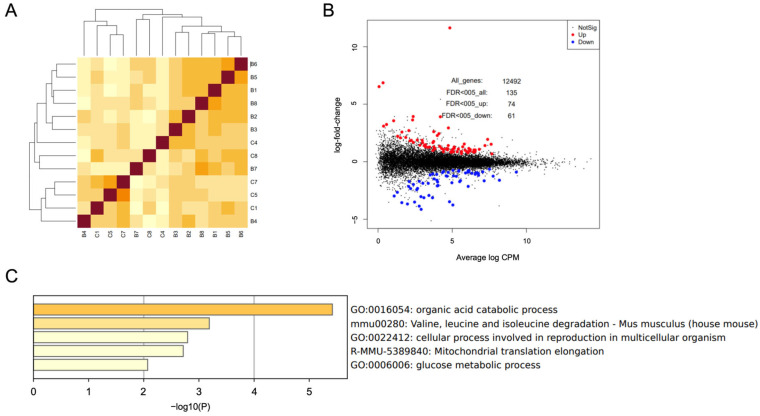
Effects of CO-UFB on the gene expression of blastocysts cultured in vitro. (**A**) Correlation heatmaps. Colors represent correlation coefficients between samples, with darker colors indicating stronger correlation. (**B**) Differentially expressed genes. (**C**) Characterization of up-regulated genes affected by CO-UFB. CO-UFB: carbon monoxide ultrafine bubble.

We performed a transcriptome analysis on blastocysts of the Control (N = 8, C1 to C8) and CO-UFB (20×) groups (N = 8, B1 to B8). Hierarchical clustering and multidimensional scaling (MDS) analysis of transcriptome data from 16 blastocysts, conducted using EdgeR, identified three samples (C2, C3, and C6) as outliers, which were subsequently excluded from further analysis (Supplementary Figure S1). The remaining 13 samples were analyzed using the EdgeR software version 4.2.2. After filtering out genes that were not expressed in either group, the count data for 12,492 genes underwent TMM normalization. A comparative analysis between the control and CO-UFB (20×) groups revealed 74 genes that were significantly upregulated in the CO-UFB (20×) group (Figure 4B). Functional enrichment analysis of these upregulated genes indicated associations with Gene Ontology (GO) terms such as “Organic acid catabolic process”, “Valine, leucine, and isoleucine degradation”, “Cellular process involved in reproduction in multicellular organisms”, “Mitochondrial translation elongation”, and “Glucose metabolic process” (Figure 4C).

## 4. Discussion

Carbon monoxide, a type of gasotransmitter, can be introduced to embryonic cells via ultrafine bubbles, facilitating the efficient differentiation of mouse oocytes into blastocysts. In this study, the optimal concentration of carbon monoxide ultrafine bubbles for promoting the differentiation of fertilized zygotes was determined to range from approximately 1 × 10^8^/mL to 6 × 10^8^/mL, with no significant effects observed at either higher or lower concentrations. Additionally, we confirmed that the UFBs remained stable within the culture medium for up to 180 days. These findings suggest that CO-UFB-enriched culture media may enhance the success rate of IVF by fostering blastocyst differentiation, potentially through the cytoprotective effects of the gasotransmitter.

NO, CO, and H_2_S are endogenous gaseous signaling molecules with distinct physiological roles. NO and CO primarily activate the soluble guanylate cyclase/cyclic guanosine monophosphate pathway, whereas H_2_S exerts anti-apoptotic and antioxidant effects through the activation of the ATP-sensitive potassium channel [39]. These gasotransmitters, when administered exogenously—either as the gases themselves or via donor compounds—serve cytoprotective functions. NO donors are already employed as crucial therapeutic agents in the management of cardiovascular diseases. Similarly, CO and H_2_S donors are actively under investigation in both animal models and human clinical studies. Given their extremely short half-lives in cell culture media, these gasotransmitters are typically delivered to cells via donor molecules. Notably, the use of gasotransmitter donors has been explored in the field of reproductive medicine. Physiological production of NO is vital for successful IVF and embryo culture. Disruption of NO regulation has been implicated in granulosa cell apoptosis, oocyte aging, and diminished embryonic differentiation capacity in vitro, as demonstrated in studies involving bovine [40] and murine [41] models. Supplementation of culture media with NO donors has been shown to mitigate apoptosis and enhance embryonic development rates in both human [42] and bovine [43] systems. Likewise, H_2_S, akin to NO and CO, plays a significant role in cell differentiation, development, and cytoprotection [44]. Inhibition of physiological H_2_S production has been associated with failure in oocyte maturation in porcine models, underscoring its critical involvement in reproductive health [45]. Additionally, supplementation of culture media with H_2_S donors has been documented to enhance embryonic development in in vitro porcine systems [46]. Regarding CO donors, the development of CO-releasing molecules (CORMs) designed to facilitate the transport of CO into aqueous solutions and cells is actively progressing, with their safety largely established, primarily based on studies in rodent models such as mice and rats [47]. Although no studies have yet reported the application of CO donors in the field of fertility treatment, like NO and H_2_S donors, they hold promise for improving embryo differentiation rates. This potential stems from CO’s ability to bind to various mitochondrial proteins, thereby activating mitochondrial functions that mitigate ROS production [48,49,50,51]. In fact, CORM has been shown to ameliorate atherosclerosis-induced symptoms by downregulating miR-34a-5p and enhancing mitochondrial function [52]. Additionally, CORM has been reported to inhibit the generation of cardiac ROS within mitochondria, thereby reducing cardiac oxidative stress [53]. Consequently, CO has been demonstrated to enhance mitochondrial function and exhibit antioxidant properties when exogenously administered to cells. In this study, genetic analysis of blastocysts revealed that exogenously introduced CO is internalized by embryonic cells and activates a subset of genes associated with mitochondrial activity. However, these donors, which contain metal complexes, present a potential concern regarding metal toxicity to cells.

Fine bubbles are characterized as bubbles with a diameter of less than 100 µm. A further classification distinguishes microbubbles, which have a diameter between 1 µm and 100 µm, from UFBs, which are smaller than 1 µm in diameter [20]. Notably, UFBs do not float in water and are typically stable without stimulation, rarely dissolving or buoying unless induced. Reports indicate that UFBs can maintain stability in liquids for several months or even longer [22]. The current study also demonstrated that CO can be encapsulated within UFBs, with sizes ranging from approximately 100 to 200 nm, allowing for long-term storage in culture media over extended periods. This unique property of fine bubbles has prompted investigations into their potential as oxygen donors, particularly in the medical field. Oxygen microbubbles have been shown to enhance pancreatic islet cell survival or improve outcomes in rat models of acute lung injury [54,55]. Similarly, oxygen ultrafine bubbles have been reported to prevent bone loss in osteoporotic mice or promote recovery from sciatic nerve crush injury [56,57]. In this study, CO, a gasotransmitter, was successfully administered to embryonic cells by exploiting the characteristics of UFBs, circumventing the issue of metal toxicity associated with CORMs. This method holds promise for improving the efficacy of current donor-based gasotransmitter delivery systems. Several mechanisms may account for the uptake of CO encapsulated in UFBs by cells. One potential mechanism involves the formation of micropores in the cell membrane as the bubble collapses near the membrane, facilitating the migration of the locally released contents into the cell [58]. Alternatively, it is plausible that UFBs may be internalized via endocytosis, given their size, which is comparable to that of exosomes [34].

This study demonstrated that the administration of CO via UFBs may enhance the differentiation potential of fertilized oocytes, which was confirmed by the genetic analysis of differentiated blastocysts. However, this study had several limitations. First, the concentration of CO within the fertilized oocytes was not directly measured, preventing a precise assessment of the extent to which it was absorbed by the fertilized oocytes. Although various methods, including gas chromatography, infrared laser spectroscopy, chemical probe-based techniques, and Hemo CD-based assays, have been employed to measure intracellular CO, their limitations—such as the need for specialized equipment and lack of accurate quantification—rendered them unsuitable for this study [59,60,61]. Importantly, CO-containing gasotransmitters can exert cytotoxic effects at high concentrations, necessitating precise dosing to ensure safety and efficacy in embryo culture systems. Moreover, UFBs exhibit rapid declines in concentration and become unstable immediately after generation. Therefore, a stabilization period is typically recommended prior to their use. In this study, CO-UFB media were intentionally used on Day 1 after generation, before full stabilization, to assess a broader range of concentrations. However, a gradual decline in UFB concentration over the culture period cannot be ruled out. This limitation should be addressed in future studies through more controlled timing and concentration tracking. Additionally, the comparisons made in this study were primarily made between each CO-UFB group and the control group. That is, we did not make any statistical comparisons between different CO-UFB concentrations (e.g., 20× vs. 100×). Future investigations should directly evaluate the dose–response relationship to better define the optimal concentration range for embryonic development.

It should also be noted that, in clinical ART settings, fertilization and oocyte maturation commonly occur in vitro. Therefore, further studies are warranted to evaluate the effectiveness of CO-UFB in supporting embryo development and implantation following IVF and embryo transfer. Despite these limitations, the method of CO administration, which does not introduce metal toxicity, holds considerable promise, analogous to the established applications of NO and H_2_S. This strategy may also be extended to other ART-related media, including those used for semen and oocyte preservation, where oxidative stress remains a critical concern. Thus, the findings offer the potential for broader advances in infertility treatment.

This study also monitored UFB persistence for up to 180 days, and their detectability over this extended period suggests they were physically stable. The concurrent decrease in particle concentration and increase in average particle size over time (Figure 1) suggest that partial coalescence of ultrafine bubbles may have contributed to these changes. While this observation does not fully cover the 12-month shelf life typical of SSCM, the intrinsic physicochemical characteristics of UFBs—such as high internal pressure and surface charge—support the possibility of longer-term stability under optimized conditions. Continued investigation is needed to clarify the long-term dynamics of UFBs in culture systems. Finally, while these results are encouraging, directly extrapolating from mouse models to human IVF applications should be approached with caution. Further validation in human-derived systems will be essential for clinical translation.

## 5. Conclusions

In conclusion, CO, when encapsulated in UFBs, can persist in the culture medium for extended durations and effectively facilitate the differentiation of fertilized mouse oocytes into blastocysts, owing to its antioxidant and cytoprotective properties. This approach holds significant potential for enhancing IVF outcomes, thereby potentially improving fertility rates. However, further research is required to determine the optimal concentration of UFBs for promoting fertilized oocyte differentiation and to comprehensively elucidate the underlying mechanisms.

## Data Availability

The datasets generated and/or analyzed during the current study are available from the corresponding author upon reasonable request.

## Supplementary Materials

The following supporting information can be downloaded at: https://www.mdpi.com/article/10.3390/antiox14060684/s1, Figure S1: Correlation heatmap illustrating the effects of CO-UFB on gene expression profiles in in vitro-cultured blastocysts.
