# Supplementary material for: Culture Medium Enriched with Ultrafine Carbon Monoxide Bubbles Enhances In Vitro Blastocyst Formation of In Vivo-Fertilized Mouse Zygotes
Source: Antioxidants (Basel). 2025 Jun 4;14(6):684. doi: 10.3390/antiox14060684 (PMC12189054; doi:10.3390/antiox14060684)
Supplement: Supplementary file 1 [file antioxidants-14-00684-s001.zip › antioxidants-3652631-supplementary.pdf]

Supplementary Figure

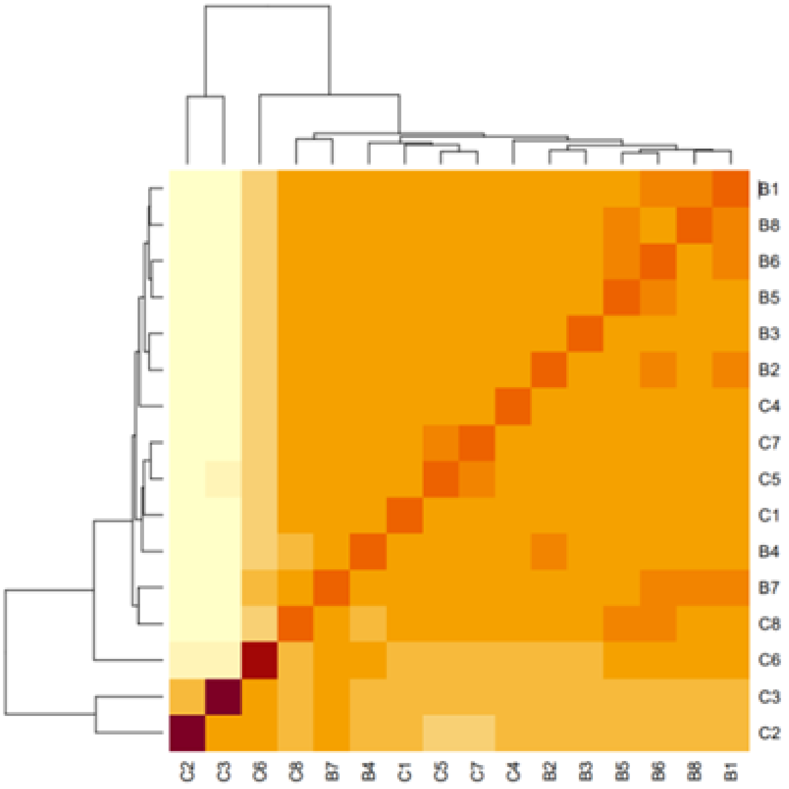

Figure S1. Correlation heatmap illustrating the effects of CO-UFB on gene expression profiles in *in vitro*-cultured blastocysts.
